# Africa and the Cattle Without History

**DOI:** 10.3828/whp.ge.63834608440709

**Published:** 2024-10

**Authors:** Tad Brown

**Keywords:** Livestock, development, genetics, Africa, history of science

## Abstract

The premise of this article is that African historiography has yet to embrace the genetic basis of cattle tolerance to tsetse-borne trypanosomiasis due to the literature’s emphasis on human illness and landscape modification. By the early 1980s, empirical research indicated that N’Dama cattle possessed a tolerance to the disease that was heritable and, as such, could be strengthened through breeding. The Gambia’s first president, who was a former veterinary surgeon, contributed to the breed’s reappraisal. In exploring this history, I show how an international scientific network positioned The Gambia as a supplier of N’Dama breeding stock for livestock developments in sub-Saharan Africa. My argument is that research on cattle genetics has theoretical consequences for writing about the history of African tsetse ecosystems.

## Herds of the fly

In the spring of 1984, a small herd of Boran cows gave birth to N’Dama calves. The two breeds belong to the different subspecies of domesticated cattle, N’Dama to *Bos taurus* and Boran to *Bos indicus*.^1^ Of the 29 Boran heifers surgically implanted with frozen embryos, eleven got pregnant. Then one aborted. Nonetheless, scientists at the International Laboratory for Research on Animal Diseases (ILRAD) in Nairobi, Kenya proclaimed the project a success. These N’Dama, conceived in The Gambia yet birthed across the continent, were a proof of concept. It was the first attempt to reproduce indigenous African cattle in the uterus of a surrogate breed. Born of another mother, the ten foster calves displayed superior immunity as compared to Boran calves in the same trial.^2^ Demand for N’Dama across sub-Saharan Africa was no longer limited by the availability of live animal exports.

In this article, I explain how veterinary knowledge of cattle genetics repositioned N’Dama, specifically from The Gambia, within scientific visions of rural development. Only a fraction of cattle in Africa can withstand a debilitating disease spread by tsetse flies (*Glossina* spp.), which inhabit roughly a third of the continent. N’Dama is one such breed.^3^ Historians have focused primarily on the human version of the tsetse-borne disease, called ‘sleeping sickness’.^4^ The equivalent of sleeping sickness in non-human populations is African animal trypanosomiasis or *nagana*, a term derived from the Zulu word for ‘depressed spirit’.^5^ Symptoms include chronic fatigue, poor growth, low milk output, abortion and fatality. Tolerance to the disease, known as trypanotolerance, is no invincibility, yet African pastoralists, commercial ranchers and veterinary scientists have long observed that certain cattle showed a marked ability to survive in tsetse ecosystems.

Tsetse disease ecology has been a central theme of African environmental history, mostly with regards to humans.^6^ The topic’s related animal history tends to be subsumed within a greater narrative of tropical medicine. In 1894, the British Army Medical Service sent David Bruce to Zululand to investigate the cause of African animal trypanosomiasis. Bruce went on to identify a group of protozoa, *Tryopanosoma*, as the biotic agent responsible for the disease. He learned that when tsetse flies take a blood meal, the vector delivers trypanosomes through its saliva into the host’s bloodstream.^7^ Numerous species exist and affect their vertebrate hosts to different degrees, but both people and domesticated animals can suffer fatality as blood-borne pathogens multiply in the body.^8^

The virulent infection accounts for severe losses to agricultural production in sub-Saharan Africa to this day.^9^ Such observations are nothing new. At the opening address to the 1925 League of Nations conference on sleeping sickness in Africa, the British parliamentary under-secretary observed: ‘From the economic, social and administrative points of view, nagana (cattle Trypanosomiasis) is as important as, if not more important than Sleeping Sickness’.^10^ Colonial scientists adopted three basic strategies for managing the livestock disease – vector control, drug therapies and breed choice.^11^ This last approach explains why N’Dama calves from West Africa would come to be nursed by Boran cows in East Africa.

Historian Samuël Coghe has recently focused on colonial grazing schemes of French Equatorial Africa that relied on trypanotolerant cattle. Commercial ranchers began to import N’Dama, as well as West African Shorthorn cattle, to the region in the late 1940s. The ventures were less concerned with the science of disease tolerance than its results: large imported cattle herds were established in the humid, tsetse-infested savanna.^12^ Earlier attempts to relocate N’Dama to Central Africa had validated the economic prospect. Trypanotolerant cattle arrived by boat to Belgian Congo (now Democratic Republic of the Congo) in 1920, with subsequent shipments to follow, providing evidence that N’Dama could be introduced and commercialised in places infested with tsetse flies.^13^ Successes varied, however. For all practical purposes, these husbandry triumphs were a consequence of capital investment, veterinary drugs, tsetse densities and breed choice.

The animals’ unknown clinical background undermined scientific confidence in the implication of these episodes. There was no way to know, in hindsight, if transplanted N’Dama had displayed an innate ability against tsetse-borne trypanosomiasis in the new environment. Scientists put forth various possible factors to explain the immune response, including the transfer of maternal antibodies.^14^ Veterinary experts had not actually ‘grasped the basic mechanisms’ of tolerance by the late 1940s, as Coghe suggested, only its general association with specific breeds.^15^ Colonial scientists tended to view cattle tolerance to trypanosomiasis as territorial in scope and that herds were only adapted to local strains. Movement of animals began to overthrow the paradigmatic belief.^16^ Sometimes N’Dama were able to withstand the disease beyond their birthplace, but how?

A understanding of trypanotolerance heritability in the 1980s brought with it far-reaching implications for development. If large-scale N’Dama exports earlier in the century represented ‘development as an experimental science’, by the late colonial period, science became viewed as the basis for making responsible economic decisions in the colonies, including with the treatment of livestock.^17^ The relationship between science and development experienced another change in the decades after political independence in Africa. Research projects became an economic opportunity in their own right.^18^ In this context, veterinary experts proposed a scientific vision for sustainable livestock production in African tsetse ecosystems. Rather than proceeding on the gross estimate of breed choice, experimental cattle breeding could, in theory, lead to greater productivity in tsetse ecosystems. The development prospect attracted the support of international funding agencies.

At the time of the embryo transfers to Kenya, trypanotolerant cattle comprised only five per cent of the 147 million cattle in African countries riddled with tsetse flies.^19^ Nonetheless, the successful experiment at ILRAD added empirical findings to the debate about whether N’Dama ability to produce milk and meat in tsetse ecosystems was an acquired trait or not. Evidence for an inherited disease tolerance redefined the scope and nature of scientific breeding with N’Dama cattle by separating the nature of disease tolerance from any specific locale. In the process, The Gambia became identified as a preferred source of N’Dama genetics and a place for research on trypanotolerance. This article presents a twentieth-century Anglophone history of veterinary knowledge about these African cattle.

After a review of the literature on tsetse control, I introduce Sir Dawda Kairaba Jawara (1924–2019) and discuss observations about cattle in Gambia and West Africa more broadly. Jawara, a one-time veterinary surgeon and first president of The Gambia, played a decisive role in identifying his country as the preferred source for N’Dama. The article demonstrates how veterinary scientists – including those from Jawara’s alma mater in Scotland – transformed the study of African animal trypanosomiasis. A few of these same scientists supported President Jawara in founding an international research centre in The Gambia dedicated to the study and promotion of trypanotolerance. Through a social history of science, I show how veterinary experts reframed Gambian N’Dama as a genetic resource for research and development in African. The article, reflecting on the findings from animal science, presents a challenge to orthodox theories about tsetse ecosystems in African historiography.

## Breeds and theories

Historians have thoroughly examined the relationship between veterinary knowledge and livestock development projects in Africa, giving special attention to disease control campaigns during colonialism.^20^ Observations about ‘native’ breeds were not simply a report of biological traits. As Wesley Mwatwara and Sandra Swart have shown, the commentary was also about who possessed which animals.^21^ Unsurprisingly, what the colonial archives reveal is that veterinary officers tended to hold a negative opinion of African cattle. Published findings of breed traits were cited repeatedly through an institutional network keyed to the same cultural cues and reference works, as the replacement of African cattle with European stock became a normative fact of imperial design.^22^ This dark underbelly of breed profiling has impacted the course of livestock development in Africa.

Sustained attention to specific breeds has rarely been a prerogative in environmental history.^23^ An allegiance to pedigree records has biased which cattle historians discuss, from where, and how far back in time. In the unforgettable words of Rebecca Woods, the history of livestock improvement depends on the stud books of ‘herds shot round the world’.^24^ N’Dama cattle, being less subject to scientific record-keeping, lack the written documents of European breeds.^25^ That pastoralists in West Africa demonstrate a knowledge of their animal breeding lines going back generations is the sort of ethnographic detail absent in most historical accounts.^26^ Instead, the presence of cattle has served as a cue in narratives of African environmental history, signaling the relative lack of tsetse flies.

Arguably more than anyone, John Ford – a trained entomologist – highlighted the failure of colonial scientists to combat the tsetse-borne disease. In *The Role of the Trypanosomiases in African Ecology*, Ford explained how the earliest methods of colonial tsetse control relied on deforestation and all-out wildlife slaughter. These efforts to deprive the vector of its habitat and food source had huge impacts, just not necessarily on tsetse.^27^ Veterinary scientists in settler colonies like South Africa, who were tasked with developing a commercial cattle industry for white landowners, resorted to late-season burning and massive land clearance to make the country uninviting to tsetse, which avoid open spaces.^28^ As with so many colonial improvement projects, the victories of tsetse control were sporadic, localised and all too temporary.

According to Ford, trypanosomiasis had intensified under colonialism. He theorised that, prior to European rule, village life in Africa had affected a low-level contact with trypanosomes because land-use intensification kept tsetse bush habitat from encroaching on agrarian settlements. Ford suspected that demographic decline, village dispersal and wildlife depopulation had resulted in a historic caesura of infection, which led to the serial outbreaks of sleeping sickness witnessed by colonial officers. If a temporary lapse of contact with the blood-sucking fly caused devastating results for African peoples, what about their cattle?

Africanist historian James Giblin charged that Ford is largely misunderstood by historians, who tend to attribute adaptation during the precolonial period to tsetse evasion, rather than ‘*limited*, though continued, exposure to infection’.^29^ Drawing from his own work on the early twentieth century, Giblin stated that people in northeastern Tanzania had replenished herds from outside the tsetse belt, buying cattle that ‘had poor prospects of developing resistance’ to trypanosomiasis.^30^ Sourcing cattle from areas free of tsetse is used by Giblin to explain subsequent losses. He attributed the cause of death to an interruption of regular prophylactic encounters with the vector.^31^ African societies and their herds were now both highly susceptible to the tsetse-borne disease.

Giblin further defended the Fordist theory of acquired resistance by rejecting its alternative, typified by the evolutionary argument of A.J. Duggan. An expert in tropical medicine, Duggan theorised that the virulent outbreaks of trypanosomiasis were evidence of a parasite ill-adapted to its host. Unlike Ford, he postulated that events of the early twentieth century were an abbreviation of a much longer adaptive cycle. To arrive at this conclusion, Duggan compared infectivity at ‘old endemic foci’, where the tsetse-borne disease had existed for years, with the human trauma experienced at sites of more recent infection. The data indicated that ‘serious local outbreaks occurred soon after its introduction and later these settled down into an overall endemic pattern of low prevalence’. Therefore, in Duggan’s view, African societies had avoided certain death in tsetse ecosystems, not from cultural mediation of infection rates, but from natural selection. Duggan saw that prolonged contact with the parasite yielded to a ‘tranquility of residence’, an immunity still pending in the long arc of commensalism.^32^

In the historiography of sleeping sickness, it is Ford’s theory – that mild infection permitted an indefinite tenancy in tsetse ecosystems – that has been applied to cattle.^33^ Historians tend to view acute outbreaks of trypanosomiasis as self-evident disturbances brought about by capitalism and colonialism.^34^ Compiling land-use histories introduces some scepticism to this claim. The idea that European rule introduced a sudden change in grazing patterns, which determined how herders and their herds interacted with tsetse flies, assumes too much about the relative freedom of previous pastoral habits. For example, Richard Waller illustrated that, at least for the Western Narok territory of Kenya Maasailand, instability was a characteristic feature of rangeland ecology in the area, long before colonial incursions.^35^ The fact is that too few traces of the *longue duree* have been unearthed to reconstruct the historical ecology of African pastoralism and its adaptations to tsetse flies.

The evolutionary position, on the other hand, has been avoided as an explanation for events of the twentieth century. And for good reason. Parasitology alone hardly explains human history.^36^ This does not mean that selection played no part in recorded events, however. Edmund Russell has convincingly argued for the need to include evolutionary forces within historical interpretations.^37^ In this way, genetics can complement the timeframe of archival methods and help make inferences about the past.^38^ This article contends that a genetic understanding of N’Dama cattle recontextualises the colonial record vis-à-vis longer evolutionary trends.^39^ Evidence of cattle trait heritability in relation to tsetse-borne infection presents a case for thinking additively about Ford’s thesis of environmental management in African historiography.

## Gambian N’Dama

Retrospective accounts from the nineteenth century testify to sleeping sickness in West Africa, endemic to the vicinity of certain waterways.^40^ Colonial reports described these areas abuzz with *Glossina* as being inhibitive to livestock husbandry. Other veterinary concerns were simply more pressing to the colonial economy. A plague-like outbreak of rinderpest – a virus with staggering mortality in cattle herds and wild game – ravaged Africa from the 1890s.^41^ Veterinary scientists eventually devised a vaccine to stop the unprofitable loss of life and eradicate the murrain. Only with the rinderpest virus in check did colonial officers turn their attention to the parasite spread by the antagonistic flies.^42^

While rinderpest swept through the continent, trypanosomiasis lived there. (As late as 1990, veterinary scientists agreed that ‘no other continent appears to be [as] dominated by one disease’ to the same extent as Africa was by trypanosomiasis.)^43^ The ubiquity of tsetse flies in West Africa, in particular, explains why colonial efforts to combat the vector rarely reached the same importance as in other parts of the continent. A futility defined the whole endeavour. Besides, certain cattle in the region could outlast the effects of trypanosomiasis, at least enough to satisfy local needs. Urban demand for beef and state-led projects to stimulate growth in the rural economy prompted an interest in breed choice during the late colonial period.^44^ Around the same time, British colonial officers started a small push to recruit Africans to the veterinary profession.

When Norman Hall, the first director of the Gambian colonial veterinary unit, retired in 1949, few Africans had worked with him as trained personnel. In fact, there had been only one. Joseph Okafor, a Nigerian, accompanied Hall to the Gambia twenty years prior to launch the vaccination campaign against rinderpest. Though colonial funds had been earmarked to recruit a Gambian for the vet school in Vom, Northern Nigeria, the post sat vacant. Hall explained the vacancy by noting, ‘Veterinary work does not appear to appeal to the African.’^45^ The Director’s comment was reflective of wider stereotypes held within colonial agricultural departments. Far from not being up to the task, it was more likely that young Gambians had other educational aspirations. The career of Dawda Jawara is a case in point. He accepted a scholarship from the Colonial Office to pursue veterinary work after foregoing his desire to study human medicine.^46^

Jawara returned home from the University of Glasgow Veterinary School in 1954, having graduated with honours – only the second West African to qualify as a veterinary surgeon.^47^ He took a post in the Gambian Veterinary Department, where its staff trekked the length of the River Gambia each dry season to inject cattle herds against rinderpest. During this disease control campaign, Jawara met with chiefs in Gambia’s rural districts, laying the foundations for his later political career.^48^ ‘Since I was a boy and the Chief Veterinary Officer in The Gambia’, Jawara would reflect, ‘I came to realize the importance of N’Dama cattle to most of the families in The Gambia and to the economy of the country.’^49^ The importance of N’Dama was made evident, in part, due to a popular riverside cattle trade.

**Figure 1:**
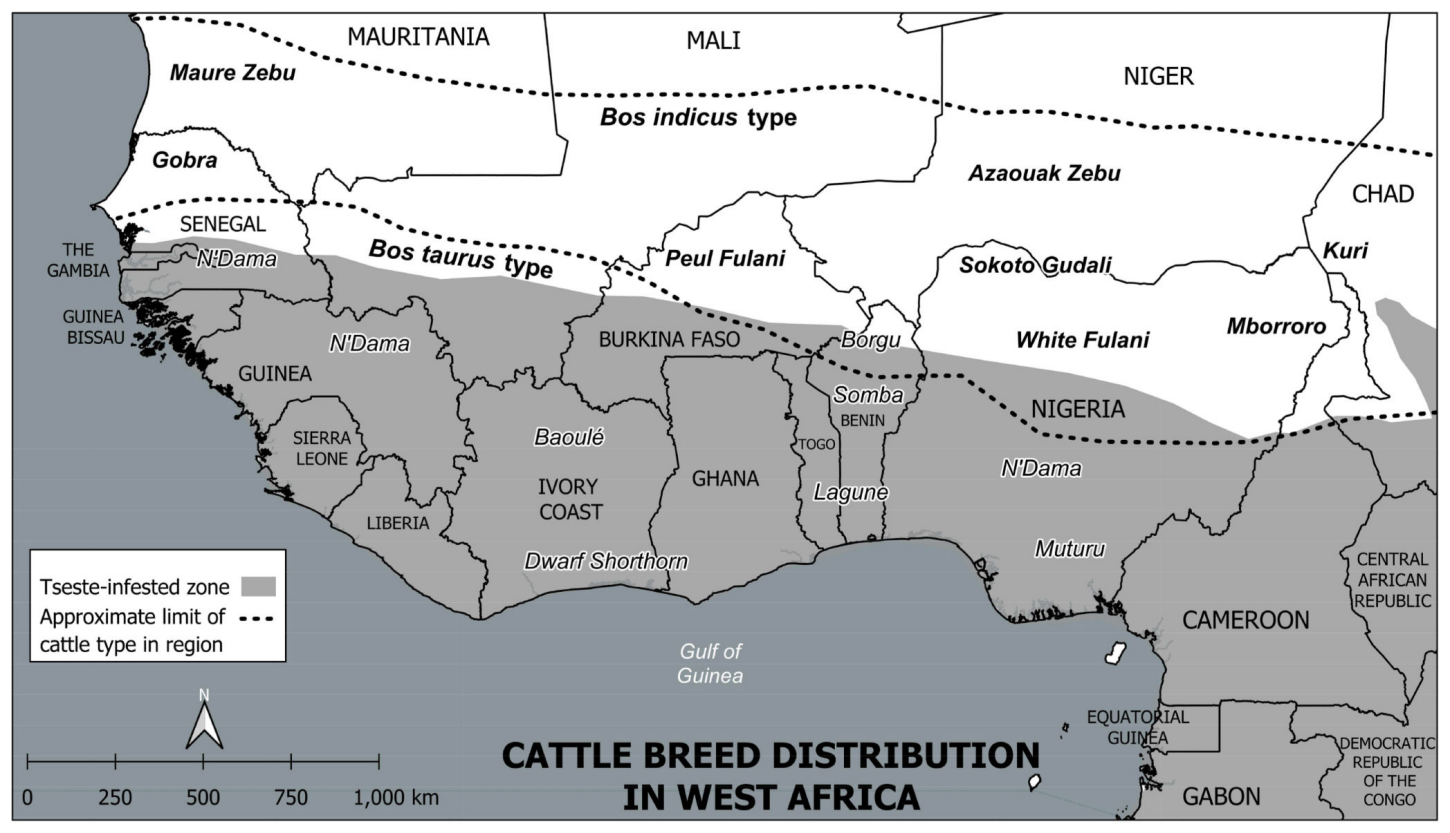
General distribution of cattle breeds relative to tsetse geography of West Africa. Adapted from Mason (1951) and Freeman et al. (2004) with updated political borders. Credit: Kelsey Lowe.

Livestock markets in Gambia drew cattle from across West Africa. What became obvious from these cattle movements, at least to those in the Gambian Veterinary Department, is that certain breeds were physically jeopardised by contact with the biting fly.^50^ Tsetse inhabited the lower stretch of the River Gambia, but not the vast arid geography of the nearby Sahel. (See Figure 1) Cattle from the interior of Senegal and French Sudan (Mali) would arrive to market emaciated with anaemia, whereas herds from humid regions to the south were accustomed to contact with *Glossina*. Gathering together cattle from across the region amounted to a comparative rural collection and provided a unique opportunity to view in one locale what was normally dispersed across the landscape.

Scientists in the field of tropical medicine had learned to define, survey and analyse insect vectors after World War I.^51^ This same focus on quantifying complex interactions ‘in the bush’ did not extend to colonial veterinary studies. Apart from the marketplace, scientists made spot observations of African cattle during seasonal rural tours. These assessments occurred without knowing how disease or environment influenced animal performance. Controlled comparisons between various types of cattle simply did not exist, and, by default, Europeans tended to disparage the native herds encountered. Empirical field studies with different breeds living under the same husbandry conditions were needed to evaluate the relative productivity of each cattle type.^52^

Classification came first. During his inaugural visit to the Gambia in the late 1920s, Norman Hall proposed that its cattle population could be ‘conveniently divided into three types, all more or less distinct’ – West African Shorthorn, N’Dama longhorn and N’Dama-Zebu cross. Hall described the dwarf West African Shorthorn as the ‘only pure breed’, although he conceded that N’Dama also exhibited fixed traits. The most significant trait of either cattle type was a tolerance to trypanosomiasis, which the Zebu-cross lacked and, what is more, threatened to undermine.^53^ Colonial officers across British West Africa would observe that breeding Zebu cattle (*Bos indicus*) with N’Dama (*Bos taurus*) seemed to increase susceptibility to trypanosomiasis in the crossbred offspring.^54^ In Gambia, the Zebu influence occurred along the trade routes from Senegal, as sale cattle bred with local herds during transport.

The third veterinary director of the Gambia, Sam Walshe, revised the classification issued by his predecessor. Walshe’s estimation in the 1950s was that ‘the local cattle are not N’Dama, nor are they a fixed breed, being made up chiefly of a mixture of N’Dama and Zebu blood with a small mixture of the blood of the now extinct dwarf cattle, the true West African Shorthorn’. According to Walshe, the phenotype of a ‘typical Gambian herd’ resembled N’Dama but did not breed true, giving rise to a range of forms. Furthermore, he doubted that the local cattle in Gambia – which he said were ‘erroneously referred to as N’Damas’ – had the same resistance to trypanosomiasis as ‘the true N’Dama’ in humid British territories to the south, where the vegetation and rainfall made tsetse flies more populous.^55^ Gambia had shared a Department of Agriculture with Sierra Leone until Walshe’s arrival, so personnel likely had first-hand experience with cattle in both colonies. The breed breakdown and its implications would be put to the test.

Under Walshe, the Gambian Veterinary Department began purchasing N’Dama cattle from Sierra Leone. The decision had its critics. Why import N’Dama when the local breed was N’Dama? Cattle from both places shared a superficial resemblance, yet observation in the Gambia seemed to confirm Walshe’s suspicion. ‘After two dry seasons’, Walshe reported, ‘the condition of the [imported] N’Damas is if anything superior to that of the local cattle.’^56^ What is more, the excellence of the relocated stock contradicted colonial scientists’ opinion that disease tolerance in cattle was based on circumscribed strains of *Trypanosoma*. N’Dama from Sierra Leone outperformed cattle in the Gambia, despite a spatial removal from their previous disease environment.

Knowledge about cattle type distribution gained importance during World War II. Facing shortages of meat and fats in the British empire, colonial officers began to conduct extensive surveys and make scientific recommendations about livestock development and disease control in Africa. T.H. Davey from the Liverpool School of Tropical Medicine visited British West Africa in 1945 to study trypanosomiasis, and P.A. Buxton toured East and Central Africa for the same purpose.^57^ Around the same time, T.A.M. Nash went to Nigeria, Gold Coast, Sierra Leone and Gambia in an attempt to update the haphazard state of knowledge about tsetse in West Africa.^58^

Nash observed that cattle owners in The Gambia had recovered from their losses to rinderpest in the early decades of the twentieth century by increasingly sourcing Zebu bulls from neighbouring territories to cross with whatever N’Dama remained. (This was because Zebu cattle proved less susceptible to rinderpest than *Bos taurus*.) Nash commented, however, that many of the Zebu replacement bulls died soon after being imported due to trypanosomiasis. Rather than champion indigenous taurine cattle, like N’Dama, Nash was convinced that tsetse eradication would enable Africans to adopt the larger Zebu breed, ‘a much finer beast than those now kept’ in his opinion.^59^

Then, in 1953, the Colonial Office published a comprehensive survey of African cattle, titled *The Improvement of Cattle in British Colonial Territories in Africa*. Its authors, D.E. Faulkner and J.D. Brown, had travelled from the Gambia to what was then known as Northern Rhodesia and back up to Kenya. They visited government stock farms and veterinary research stations in each of the British colonies, along with some private ranches. Unlike regional markets beside the River Gambia, with dissimilar herds tethered side-by-side, Faulkner and Brown had to traverse huge distances to appraise the cattle diversity of British colonial Africa. The cross-continental survey indicated that the practice of upgrading local cattle with Zebu or imported breeds had some episodic triumphs, notably in the ‘European areas’ of Kenya and Tanganyika.^60^ A different fate awaited the same cattle when introduced to the rest of tropical Africa. Faulkner and Brown wrote:

It is now being appreciated that deterioration of livestock as manifested by diminished size, poor calf crops, mediocre production, susceptibility to disease and lack of vigour is not due merely to the vagaries of nature but that the fundamental cause is to be found in the inability of the animal to adapt itself to its environment.

The environment could be altered to some degree, but so could the animal. Of the colonial experts queried, the ‘almost universal opinion’ was that, second only to changes in cultural practice, ‘the selective breeding of the indigenous types’ was the most practicable method for increasing milk and meat production in tropical territories.^61^ Tolerance to trypanosomiasis took priority in the scientific recommendations for breeding livestock for West Africa.

**Figure 2:**
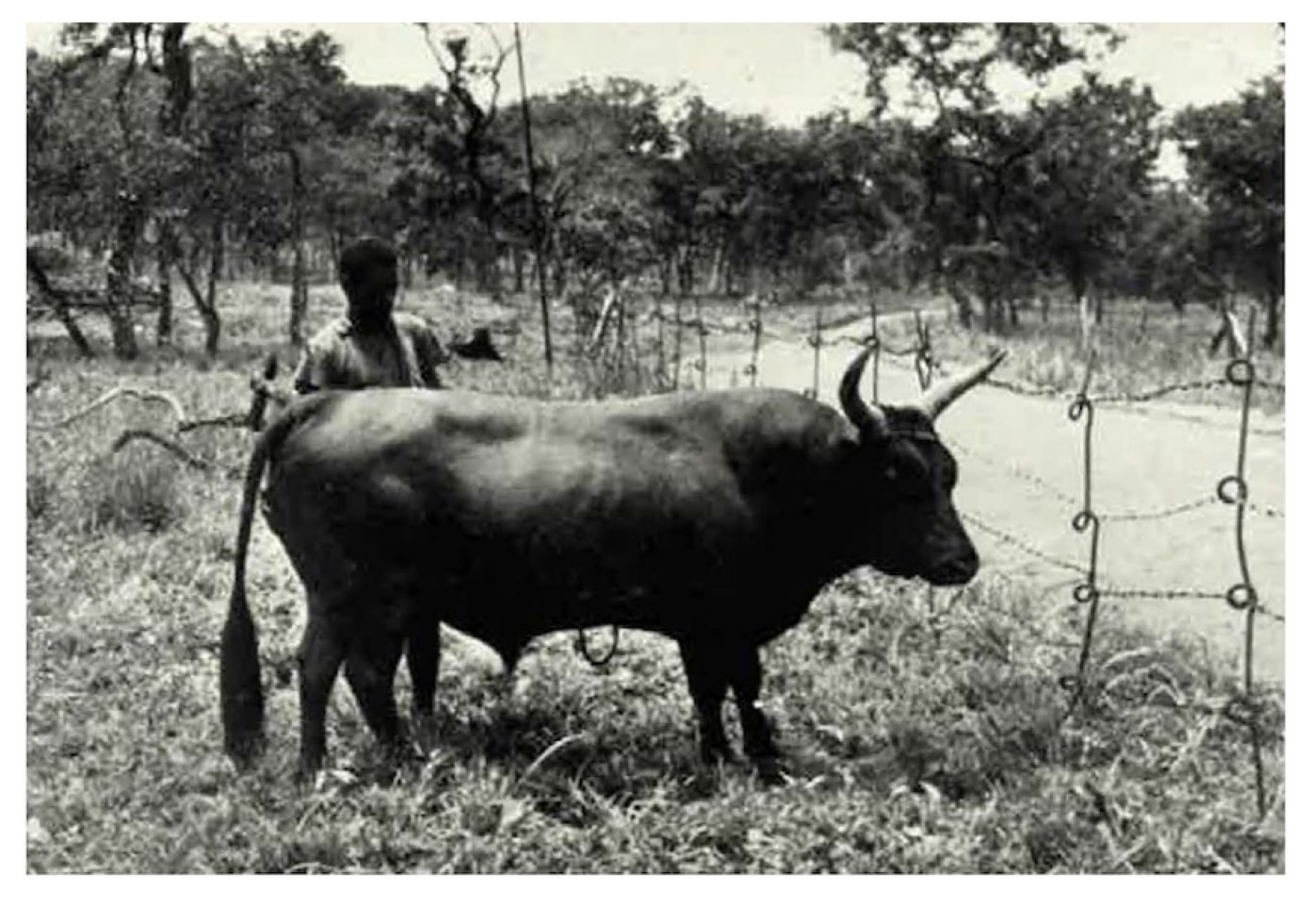
Photograph of N’Dama bull. Credit: Faulkner and Brown (London: Her Majesty’s Stationery Office, 1953).

The survey by Faulkner and Brown seemed to validate Walshe’s opinion of Gambian N’Dama. They reported on N’Dama in Sierra Leone, Nigeria and Gold Coast yet characterised Gambian cattle as ‘slightly larger than pure N’Dama’ and less tolerant to tsetse-borne disease, hinting at the influence of Zebu.^62^ (See Figure 2.)

Classification of West African cattle alerted specialists to the breed labels applied in British territories. Differences in usage flagged term ambiguity as well as gradation in the make-up of African cattle herds – with humped Zebu influence in the arid north converging with dwarf Shorthorn from the coastal forests. An intermediate type might be identified as N’Dama in Gambia, whereas elsewhere the same animal would be called something else altogether.^63^ African cattle surveys were helping to expose the shortcomings of breed typology. Still, the taxonomic activity was deemed useful for administrative purposes, adding a level of detail to government surveys.

Jawara began his service to the Gambian Veterinary Department in 1954, just after the Governor had introduced the idea of a Cattle Marketing Board to buy excess animals from upriver to sell for slaughter in the capital.^64^ At the time, the government sat on a proposal to merge the new Veterinary Department into the Department of Agriculture. Jawara vocally opposed the merger. He saw animal husbandry and veterinary medicine as a unified front, deserved of its own status with a budget to match. Fully aware that Gambians returning from university would seek employment in the civil service, he petitioned for increased wages and additional recruits.^65^ Those in charge of colonial finances scrutinised his appeal with disapproval. Fed up, Jawara retired from the Veterinary Department in 1960 to accept his candidacy for the People’s Progressive Party in the first ever general election.

Following independence in 1965, Jawara stayed in power and won the presidency.^66^ Those in The Gambia felt it ‘timely to examine the possibilities’ of breed change for ‘perhaps the control of trypanosomiasis [was] not too far away’.^67^ Agricultural officers considered importing bull semen from Britain, particularly the Red Poll, as it had contributed to the successful formation of the Senepol breed in the West Indies. Pamphlets of Santa Gertrudis from King Ranch in Texas also appear in the Gambian National Archives. During his presidency, Jawara liaised with contacts from his veterinary studies in Scotland and checked this impulse to replace N’Dama with imported cattle.^68^ In so doing, an international veterinary network recast Gambian N’Dama as a desirable genetic donor for livestock research and development in sub-Saharan Africa. This possibility depended, firstly, on understanding the nature of trypanotolerance as separable from an animal’s immediate surroundings.

## Post-independence parasitology

Cattle immunity to trypanosomiasis became subject to systematic study within the changing context of political independence in Africa. In 1963, Ian McIntyre, a professor in the Veterinary School at the University of Glasgow, was seconded to the University of East Africa in Nairobi. Having taught at Glasgow since 1951 – during Jawara’s studies – McIntyre would serve as dean in Nairobi for the next four years, developing the Faculty of Veterinary Science.^69^ McIntyre did not go to Nairobi alone. A recent veterinary school graduate from the University of Glasgow named Max Murray accompanied him to serve as a consultant to the Kenyan Game Department.^70^ McIntyre would welcome other colleagues from Scotland to study parasitic diseases, forming a group that ‘laid the foundation for modern parasitology’.^71^ A new clinical understanding of cattle disease tolerance emerged in the process.

McIntyre devoted his energies to teaching a generation of up-and-coming veterinary students, while also studying the problem of trypanosomiasis. It had long been observed that N’Dama cattle could survive in areas dense with tsetse flies. First reported in 1906, sporadic fieldnotes about the alleged immunity of the African cattle appeared in print the following decades.^72^

Loss of physique was common in infected cattle herds, but in the 1950s, a study from the Gold Coast (Ghana) found that ‘spontaneous recovery is the rule’ with N’Dama.^73^ A series of clinical studies from Northern Nigeria presented convincing evidence that N’Dama had an innate tolerance to *Trypanosoma*, including strains not found in the immediate geographic vicinity.^74^ Despite these findings, a verified explanation for the cattle’s biological response eluded scientists. Subsequent experiments demonstrated that levels of disease tolerance in crossbred herds could be improved with the introduction of selected West African breeding stock. Speculation mounted that ‘[t]he immunity of N’Dama cattle to trypanosomiasis might have a genetic basis’.^75^ Scientific testing of such as hypothesis would require greater specificity.

The term ‘trypanotolerance’ became used to describe ‘animals which are able to survive in tsetse infested areas without the aid of chemotherapy’.^76^ Yet researchers did not know how to quantify the trait. The question ‘what precisely is trypanotolerance’ proved difficult to answer.^77^ Field-testing of infected animals led Murray and McIntyre to identify two indices for monitoring the progress of trypanosomiasis: (1) parasite levels in the bloodstream, and (2) degree of anaemia, as indicated by number of red blood cells per unit volume. Trypanotolerant cattle were either able to contain the parasite or its effects. Of the two, anaemia became ‘the key marker in evaluating the status and severity of the disease in any one particular animal.’^78^ Whereas some cattle could recover from the anaemic state, susceptible hosts never did and eventually died. The most critical finding from these experimental studies was that some individual animals repeatedly demonstrated a superior ability to resist the effects of trypanosome infection.^79^

In the early 1970s, President Jawara urged McIntyre to visit The Gambia and investigate the problem of trypanosomiasis. Nearly twenty years had passed since Jawara graduated from his alma mater, and political independence had inaugurated new research opportunities in Africa. The Rockefeller Foundation funded a trip in 1973 for McIntyre and staff from the Glasgow Veterinary School to go to The Gambia. They visited again in 1974.^80^ These sponsored visits laid the groundwork for future collaboration. Around this time, Max Murray, who had since become a senior lecturer in veterinary pathology at the University of Glasgow, returned to Kenya to joined ILRAD. He began to conduct post-mortem exams on cattle in the East African tsetse ecosystems.^81^ These investigations in East Africa would prove valuable to the scientific reappraisal of Gambian cattle on the other side of the continent.

Several laboratory techniques were then in vogue for diagnosing trypanosomiasis. In 1977, Murray and his coauthors published an article in the *Transaction of the Royal Society of Tropical Medicine and Hygiene* in which they outlined an improved method for viewing the protozoa with a microscope by enhancing the visibility of trypanosomes in the uppermost ‘buffy coat zone’ of a centrifuged blood sample. It was slower by a few hours than foregoing immunological tests but more reliable, with the added advantage of being able to distinguish trypanosome species based on size and locomotion. Plus, scientists could use the same blood sample for diagnosing anaemia, even during fieldwork.^82^ Extensive studies across Africa, including those by ILRAD and scientists from Glasgow, established the accuracy of the method.

During the same period, John Trail, a colleague in Nairobi at the nearby International Livestock Center for Africa (ILCA), began doing performance testing on cattle breeds at private ranches in Kenya.^83^ His fieldwork included testing at Kilifi Ranch, a coastal sisal plantation of 2,500 hectares with a herd of 800 breeding cows. The ranch was run by a former submarine commander, who decided to stock his Kenyan plantation with Sahiwal cattle (*Bos indicus*) as well as Ayrshire cattle, a Scottish *Bos taurus* breed.^84^ The crossbred cattle at Kilifi ranch embodied a long-term breeding experiment under intense tsetse pressure. The ranch manager used prophylactic drugs to combat trypanosomiasis in the cattle, yet data gathered by Trail showed a substantial correlation between breeding history and disease response.^85^ Animals reacted to drug treatments with different effect because of their bloodlines.

In a subsequent landmark study, Trail compiled performance data across West and Central Africa to determine breed performance. He found that the West African breeds, including N’Dama, were nearly as productive as other breeds in areas with low tsetse risk. Trail’s interpretation overturned the standing scientific opinion of these smallish cows. The typical affiliation of trypanotolerant breeds with lower production and Zebu cattle with higher production reflected the conditions of husbandry and exposure to trypanosomiasis, more so than genetics. The added medical costs with Zebu cattle caused Trail and others to question if the breeding of trypanotolerant cattle could be used to counteract resistance to the few available trypanocides.^86^ The old argument for improving indigenous types of African cattle now had a strong quantitative basis.

More testing of breed differences under varied tsetse exposure helped livestock scientists to understand N’Dama vis-à-vis other cattle breeds. Throughout the 1970s, ILCA and ILRAD sponsored investigations into the potential of trypanotolerance for rural economic development. In one experiment from The Gambia, ten N’Dama cows and ten Zebu cows were entered into a village grazing system. Neither group of cattle had prior infection with trypanosomiasis, as known by historical records and parasitological screenings. The field study resulted in total mortality for the Zebu. ‘All deaths’, the scientists stated, ‘were attributable to trypanosomiasis and this was confirmed at necropsy’. On the other hand, N’Dama red blood cell volume showed remarkable durability over the course of the study in all but nursing cows.^87^

This research from The Gambia demonstrated that trypanotolerance was the clearly result of genetic differences between the cattle breeds and not acquired through early-life exposure. More importantly, the monitoring of parasite levels during the study revealed varied levels of susceptibility within the N’Dama herd. Certain animals exhibited an advantageous disease response. Veterinary scientists believed that selection could strengthen the trypanotolerant trait within breeding lines and enhance the ability of cattle to self-cure against tsetse-borne infection.^88^

## Backing the right cow

Support for genetic disease tolerance as a livestock development strategy in sub-Saharan Africa gained momentum at a time when policymakers began promoting sustainability. In 1980, the Gambian Livestock Marketing Board started buying hundreds of head of cattle each month to satisfy the demand for N’Dama breeding stock. A delegation of the Western Livestock Company from Nigeria visited The Gambia, signing a contract for 5,000 cattle over the next five years. Over 1,500 head of N’Dama were airlifted by the Agro-Gabon Company. Liberia, Ghana, even Sierra Leone now secured cattle from the Gambia.^89^ In a parallel to the historical breed dynamics following rinderpest, Gambian elders recalled that removal of cattle by the Livestock Marketing Board led to local decline of the N’Dama in some upriver districts of The Gambia.^90^

When the United Nations Food and Agriculture Organization (FAO) announced plans to establish a pan-African research institute for studying trypanosomiasis, Jawara pledged his support. In March 1981, the Gambian president returned from a trip to Italy and announced The Gambia as a prospective host, referencing the prior work on N’Dama cattle by veterinary scientists from Glasgow, ILRAD and ILCA. The Rockefeller Foundation sponsored a meeting in Bellagio later that year where Jawara, ‘preaching to the long-converted’ (including Max Murray, John Trail, and Ian McIntyre), promoted his country as a site for the new institute: its ‘10,000 cattle within easy reach of the capital’, various tsetse densities throughout the upriver districts, and the nearby proximity of Zebu cattle in Senegal.^91^ The ongoing sale of N’Dama by the Gambian Livestock Marketing Board had demonstrated the country’s capacity to assist other African countries in sourcing trypanotolerant cattle genetics.

**Figure 3:**
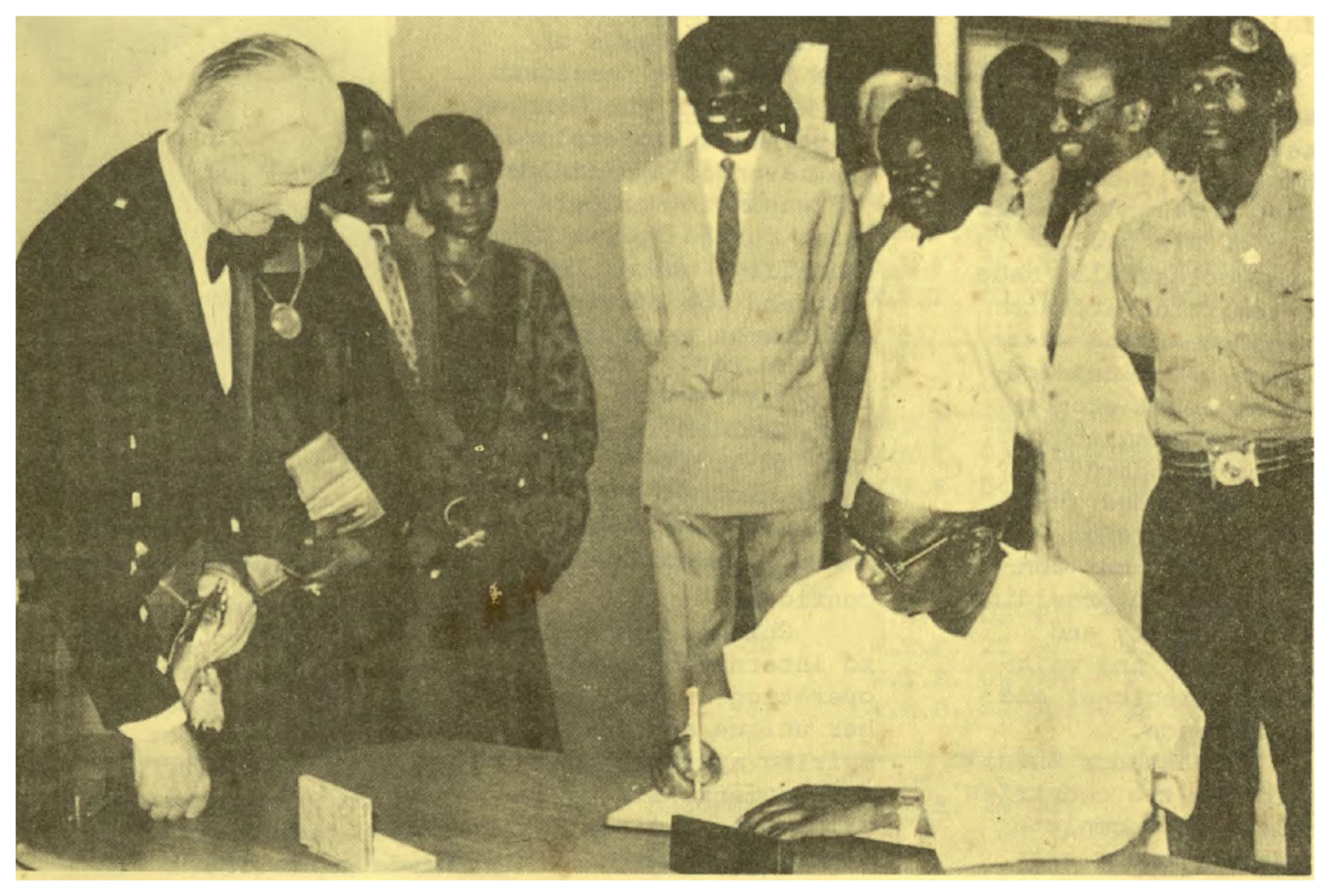
Photograph from official opening of ITC headquarters, with Director McIntyre in full regalia looking on as President Jawara signs visitors’ register. Credit: *The Gambia News Bulletin*, 1 April 1987. Reproduced with permission from Gambian National Archives.

The Gambia won the bid to host the International Trypanotolerance Center (ITC), established by an act of parliament on the last day of 1982. Three years later, ITC became fully operational with financing from The Gambia, the African Development Bank, FAO, the Rockefeller Foundation, and a host of foreign governments. Jawara chaired the inaugural council meeting to review ITC’s accomplishments under its director, Ian McIntyre, who led ITC from 1984 to 1989.^92^ (See Figure 3.)

**Figure 4:**
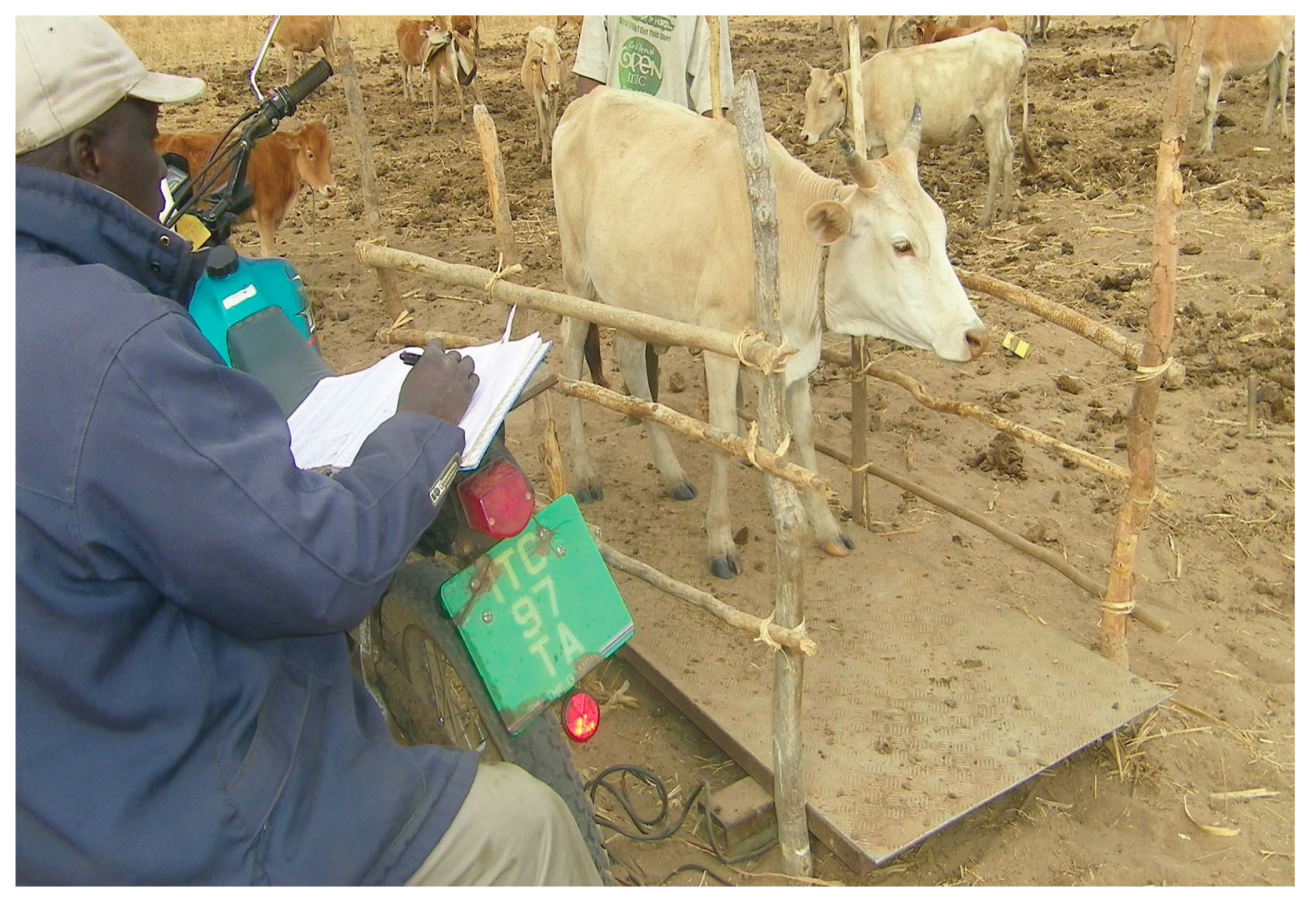
Photograph of Ima Bojang weighing N’Dama calves at ITC field site in Central River Region, The Gambia. Credit: Photo by author, 2011.

Comparative studies by researchers at ITC focused on livestock breed susceptibility to parasites. The effects of diet on immunity to *Trypanosoma* proved a key finding. Also, research at field sites across The Gambia investigated how seasonal patterns, breeding objectives and household decisions influenced the profitability of animal ownership under different levels of tsetse challenge.^93^ Since 1995, ITC has subjected N’Dama to a structured breeding programme. Animals that display a pronounced ability to gain weight and produce milk despite trypanosomiasis infection are moved to a disease-free site to reproduce. The progeny are then returned upriver where tsetse densities are high to rerun the experiment, generation after generation.^94^ (See Figure 4.) Other development agencies have joined the effort to conserve African breeds and push livestock evolution in the direction of greater disease tolerance.^95^

Reflecting on the failures of tsetse control, John Ford asked, ‘Have we backed the wrong horse?’^96^ The figure of speech could just as readily be posed for cattle. Compared to European breeds, livestock scientists have only recently begun to ask how the genotypes of African cattle could be researched for the sake of African husbandry systems.^97^ Experimental studies at ITC gave confidence to the proposition that controlled breeding could produce an animal with a better biological capacity to respond to tsetse-borne infection. Rather than import exotic stock or export any old N’Dama cow, scientists envisioned a different approach for sub-Saharan Africa. Breeding cattle with a heightened immune response to trypanosomiasis would take time and exposure, but evolution had already provided a sense of direction.

To conclude, N’Dama DNA provides insight into the *longue duree* of tsetse ecosystems. Livestock scientists have come to express ‘little doubt that the long contact of the *Bos taurine* breeds in West Africa with the parasite has been the drive for selection’.^98^ Among the results are cattle with an inherited biological advantage for surviving in tsetse ecosystems. This understanding fits awkwardly with existing tsetse narratives in African environmental history. In fact, the genetic basis to trypanotolerance challenges the reach of the Fordist paradigm.^99^ That disease tolerance evolved in West African cattle suggests more than a careful regulation of host-vector contact through landscape manipulation. N’Dama cattle display a ‘tranquility of residence’ that supports Duggan’s evolutionary perspective.

Accounting for disease pressure in breed formation does nothing to discredit Africans’ agency or local knowledge. Quite the contrary. Pastoralists are known to observe natural differences in breeding lines and select to accentuate fitness over time.^100^ Even today, herders in West Africa adapt the breed profile of their herds to exploit changes in husbandry conditions.^101^ It is certain that African pastoralists observed differences between animals and selected with a mind toward prevailing conditions. In this sense, twentieth-century scientists saw future prospects for improving disease control in animals already adapted to tsetse pressure by African herders. Defining trypanotolerance with quantitative metrics established the scientific basis for breeding to increase N’Dama cattle’s innate biological response to trypanosome infection.

The hope that animal genetic resources in Africa may possess an ‘answer’ to trypanosomiasis took hold in the decades after political independence.^102^ Geneticists have more recently noted that ‘N’Dama displays clear genetic differences compared to other African cattle’.^103^ The breed also features in arguments about the need to conserve *Bos* diversity in West Africa.^104^ The issue at stake is not whether trypanotolerance is a breed trait per se, but that it is heritable, correlated with the distribution of tsetse flies, and differs between populations and individuals.^105^ What selective breeding has contributed to sustainable livestock production in African tsetse ecosystems, other than funded research projects, is a topic requiring further study.^106^

By the early 1980s, livestock scientists had empirical evidence for a genetic basis to trypanotolerance. The small foster herd at ILRAD demonstrated that N’Dama genotypes could be reproduced within the uteruses of susceptible cows. As I have shown, these N’Dama embryos came from The Gambia because of a social history associating the place with the breed. The small West African country holds a broader significance for investigating how institutes and actors have changed the genetic landscape. What is unique about this particular instance is that few politicians have a career in veterinary medicine. Research on livestock tolerance to trypanosomiasis created economic opportunities in The Gambia because of Jawara’s education and international scientific network.

Studies of trypanotolerance led to a historic reversal of scientific opinion about the ‘local’ African breeds. The findings had consequences for both development practice and cattle population dynamics in sub-Saharan Africa. Any contention that evolutionary parasitology ‘stands outside human history and beyond the control of societies’ overlooks how the contingencies of history make genes drift and flow.^107^ Human preference alone fails to account for differentiation within *Bos*, yet in the end, there are no cattle without history.

## Footnotes

1 Crossbreeding between the distinct lineages is commonplace in Africa, where the success of pastoralism has depended on it. D.G. Bradley and D.A. Magee, ‘Genetics and the origins of domestic cattle’, in M.A. Zeder, D. Bradley, E. Emshwiller and B.D. Smith (eds), *Documenting Domestication* (Berkeley: University of California, 2006), pp. 317–28; K. Kim, T. Kwon, T. Dessie, D.A. Yoo, O.A. Mwai, J. Jang, S. Sung, S.B. Lee, B. Salim, J. Jung, H. Jeong, G.M. Tarekegn, A. Tijjani, D. Lim, S. Cho, S.J. Oh, H-K. Lee, J. Kim, C. Jeong, S. Kemp, O. Hanotte and H. Kim, ‘The mosaic genome of indigenous African cattle as a unique genetic resource for African pastoralism’, *Nature Genetics* **52** (10) (2020): 1099–110; A. Talenti, J. Powell, J.D. Hemmink, E.A.J. Cook, D. Wragg, S. Jayaraman, E. Paxton, E.T. Obishakin, E.R. Agusi, A. Tijjani, K. Marshall, A. Fisch, B.R. Ferreira, A. Qasim, U. Chaudhry, P. Wiener, P. Toye, L.J. Morrison, T. Connelley and J.G.D. Prendergast, ‘A cattle graph genome incorporating global breed diversity’, *Nature Communications* **23** (910) (2022): 1–14.

2 T. Jordt, G.D. Mahon, B.N. Touray, W.K. Ngulo, W.I. Morrison, J. Rawle and M. Murray, ’Successful transfer of frozen N’Dama embryos from the Gambia to Kenya’, *Tropical Animal Health Production* **18** (1986): 65–75. For further studies on the same animals, see J. Naessens, D.J. Grab and M. Sileghem, ‘Identifying the mechanisms of trypanotolerance in cattle’, in S.J. Black and J.R. Seed (eds), *The African Trypanosomes. World Class Parasites, Volume 1* (Boston: Springer, 2002), pp. 97-111.

3 Other *Bos taurus* cattle can self-cure from the disease, including a number of dwarf West African Shorthorn breeds, such as Baoulé, Lagune and Muturu. N’Dama attracted interest, in part, due to its relative size advantage versus these comparable breeds from the humid forest zone of West Africa. See, M. Murray, J.C.M. Trail and G.D.M. D’Ieteren, ‘Trypanotolerance in cattle and prospects for the control of trypanosomiasis by selective breeding’, *Revue Scientifique et Technique (International Office of Epizootics)* **9** (1990): 369–86.

4 For a select sample, see M.C. Musambachime, ‘The social and economic effects of Sleeping Sickness in Mweru-Luapula 1906–1922’, *African Economic History* **10** (1984): 151–73; M. Lyons, ‘Sleeping Sickness in the history of Northeast Congo (Zaire)’, *Canadian Journal of African Studies* **19** (1985): 627–33; M. Worboys, ‘The comparative history of sleeping sickness in east and central Africa, 1900–1914’, *History of Science* **32** (March 1994): 89–98; I. Maudlin, ‘African trypanosomiasis’, *Annals of Tropical Medicine & Parasitology* **100** (2006): 679–701; D.R. Headrick, ‘Sleeping Sickness epidemics and colonial responses in East and Central Africa, 1900–1940’, *PLOS Neglected Tropical Diseases* **8** (April 2014): e2772; J.M. Jackson, ‘Coercion and dissent: sleeping sickness “concentrations” and the politics of colonial authority in Ulanga, Tanganyika’, *The Journal of African History* **63** (1) (2022): 37–54.

5 K. Brown, ‘From Ubombo to Mkhuzi: disease, colonial science, and the control of *nagana* (livestock Trypanosomosis) in Zululand, South Africa, c. 1894–1953’, *Journal of the History of Medicine and Allied Sciences* **63** (July 2008): 285–322.

6 G.H. Maddox, ‘Africa and environmental history’, *Environmental History* **4** (2) (1999): 162–67; W. Beinart, ‘African history and environmental history’, *African Affairs* **99** (2000): 269–302; V.C. Kwashirai, ‘Environmental history of Africa’, *Eolss Unesco Publication* (2012); J. Carney and H. Rangan, ‘Situating African agency in environmental history’, *Environment and History* **21** (Feb. 2015): 1–13.

7 D. Bruce, ‘The Croonian lectures on trypanosomes causing disease in man and domestic animals in Central Africa’, *The British Medical Journal* (1915): 91–97.

8 In Africa, *T. vivax* and *T. congolense* infect livestock, while *T. brucei* has three subspecies; *T.b. brucei* affects cattle, and *T.b. gambiense* and *T.b.rhodesiense* cause sleeping sickness in humans. For a detailed case study, see W.F. Snow, T.J. Wacher and P. Rawlings, ‘Observations on the prevalence of trypanosomiasis in small ruminants, equines and cattle, in relation to tsetse challenge, in The Gambia’, *Veterinary Parasitology* **66** (1996): 1–11.

9 P.M. Kristjanson, B.M. Swallow, G.J. Rowlands, R.L. Kruska and P.N. de Leeuw, ‘Measuring the costs of African animal trypanosomosis, the potential benefits of control and returns to research’, *Agricultural Systems* 59 (1999): 79–98; M. Alsan, ‘The effect of the tsetse fly on African development’, *American Economic Association* **105** (1) (2015): 382–410; A. Kargbo, E. Jawo, A.I. Amoutchi, H. Koua, R. Kuey, Z. Dabre, A. Bojang and R.F.C. Vieira, ‘Knowledge, attitude, and practice of livestock owners and livestock assistants towards African trypanosomiasis control in The Gambia’, *Journal of Parasitology Research* **3379804** (2022).

10 Quoted in F.E. Nkwam, ‘British medical and health policies in West Africa c 1920–1960’ (Unpublished Ph.D. thesis, University of London, 1988), p. 66.

11 No vaccine exists for the disease. J.J. Doyel, S.K. Moloo and N.K. Borowy, ‘Development of improved control methods of animal trypanosomiasis: a review’, *Preventative Veterinary Medicine* **2** (1984): 43–52; M. Murray and J.C.M. Trail, ‘Comparative epidemiology and control of trypanosomes’, *International Journal for Parasitology* **17** (2) (1987): 621–27; S.J. Black, ‘Control of pathogenesis in African animal trypanosomiasis: A search for answers at ILRAD, ILCA and ILRI, 1975–2018’, in J. McIntire and D. Grace (eds), *The Impact of the International Livestock Research Institute* (Nairobi: ILRI and Wallingford: CABI, 2020), pp. 103–147.

12 S. Coghe, ‘A new pastoral frontier: Colonial development, environmental knowledge, and the introduction of the trypanotolerant cattle in French Equatorial Africa, 1945–1960’, *Environmental History* **27** (4) (2022): 692–721.

13 M. Murray, J.C.M. Trail, C.E. Davis and S.J. Black, ‘Genetic resistance to African trypanosomiasis’, *The Journal of Infectious Diseases* **149** (3) (1984): 311–19; Coghe, ‘A new pastoral frontier’.

14 See R.S. Desowitz, ‘Studies on immunity and host-parasite relationships: I.—The immunological response of resistant and susceptible breeds of cattle to trypanosomal challenge’, *Annals of Tropical Medicine and Parasitology* **53** (3) (1959): 293–313; C.J. Roberts and A.R. Gray, ‘Studies on trypanosome-resistance cattle. II. The effect of trypanosomiasis on N’Dama, Muturu and Zebu cattle’, *Tropical Animal Health and Production* **5** (1973b): 220–33; M. Murray, J.C.M. Trail and J.G. Grootenhuis, ‘Trypanotolerant livestock: potential and future exploitation’, *Outlook on Agriculture* **13** (1) (1984): 43–51; M. Murray and G.M. Urquhart, ‘Immunoprophylaxis against African trypanosomiasis’, in *Immunity to Blood Parasite of Animals and Man* (New York: Plenum Press, 1977), pp. 211–13.

15 Coghe, ‘A new pastoral frontier’, 699. The genetic mechanism enabling some animals to escape the immunosuppression of the disease is highly complex and is still being investigated. O. Hanotte, Y. Ronin, M. Agaba, P. Nilsson, A. Gelhaus, R. Horstmann, Y. Sugimoto, S. Kemp, J. Gibson, A. Korol, M. Soller and A. Teale, ‘Mapping of quantitative trait loci controlling trypanotolerance in a cross of tolerant West African N’Dama and susceptible East African Boran cattle’, *PNAS* **100** (13) (2003): 7443–48; H. Noyes, A. Brass, I. Obara, S. Anderson, A.L. Archibald, D.G. Bradley, P. Fisher, A. Freeman, J. Gibson, M. Gicheru, L. Hall, O. Hanotte, H. Hulme, D. McKeever, C. Murray, S.J. Oh, C. Tate, K. Smith, M. Tapio, J. Wambugu, D.J. Williams, M. Agaba and S.J. Kemp, ‘Genetic and expression analysis of cattle identifies candidate genes in pathways responding to *Trypanosoma congolense* infection’, *PNAS* **108** (22) (2011): 9304–09; A. Smetko, A. Soudre, K. Silbermayr, S. Müller, G. Brem, O. Hanotte, P.J. Boettcher, A. Stella, G. Mészáros, M. Wurzinger, I. Curik, M. Müller, J. Burgstaller and J. Sölkner, ‘Trypanosomiasis: Potential driver of selection in African cattle’, *Frontiers in Genetics* **6** (2015): 137; M. Yaro, K.A. Munyard, M.J. Stear and D.M Groth, ‘Combatting African animal trypanosomiasis (AAT) in livestock: The potential role of trypanotolerance’, *Veterinary Parasitology* **225** (2016): 43–52; S-J. Kim, S. Ka, J-W. Ha, J. Kim, D.A. Yoo, K. Kim, H-K. Lee, D. Lim, S. Cho, O. Hanotte, O.A. Mwai, T. Dessie, S. Kemp, S.J. Oh and H. Kim, ‘Cattle genome-wide analysis reveals signatures in trypanotolerant N’Dama’, *BMC Genomics* **18** (371) (2017).

16 An early description of this line of thought can be found in J.J. Simpson, ‘Entomological research in British West Africa. IV. Sierra Leone’, *Bulletin of Entomological Research* **4** (3) (1931): 165; however, J.L Stewart noted that N’Dama possess resistance to protozoa in new habitats, whereas the West African Shorthorn did not. See J.L. Stewart, ‘The cattle of the Gold Coast’, *The Veterinary Record* **41** (49) (1937): 1289–97.

17 C. Bonneuil, ‘Science and state building in late colonial and postcolonial Africa, 1930–1970’, *Osiris* 15 (2001): 258–81; J.M. Hodge, ‘Science, development, and empire: The colonial advisory council on agriculture and animal health, 1929–1943’, *The Journal of Imperial and Commonwealth History* 30 (2002): 1–26.

18 On this point, see J. Ferguson, *The Anti-Politics Machine: ‘Development’, Depoliticization, and Bureaucratic Power in Lesotho* (Minneapolis: University of Minnestoa Press, 1994).

19 ILCA, *Trypanotolerant Livestock in West & Central Africa, Volume 1: General Study* (Addis Ababa: ILCA, 1979).

20 D.L. Hodgson, ‘Taking stock: State control, ethnic identity and pastoralist development in Tanganyika, 1948–1958’, *The Journal of African History* **41** (2000): 55–78; H. Tilley, *Africa as a Living Laboratory: Empire, Development, and the Problem of Scientific Knowledge, 1870–1950* (Chicago: University of Chicago Press, 2011); W. Beinart, K. Brown and D. Gilfoyle, ‘Experts and expertise in colonial Africa reconsidered: Science and the interpretation of knowledge’, *African Affairs* **108** (432) (2009): 413–33; W. Mwatwara, ‘“Even the calves must dip”: East Coast Fever, Africans and the imposition of dipping tanks in Southern Rhodesia, c. 1902–1930’, *South Africa Historical Journal* **66** (2014): 320–48.

21 W. Mwatwara and S. Swart, “‘Better breeds?’ The colonial State, Africans and the cattle quality clause in Southern Rhodesia, c. 1912–1930,” *Journal of Southern African Studies* **42** (2) (2016): 333–50.

22 S. Krätli, ‘Cows who choose domestication: generation and management of domestic animal diversity by WoDaaBe pastoralists (Niger)’ (unpublished Ph.D. thesis, University of Sussex, 2007).

23 For examples, see S. White, ‘From globalized pig breeds to capitalist pigs: A study in animal cultures and evolutionary history’, *Environmental History* **16** (2011): 94–120; J. Specht, ‘The rise, fall, and rebirth of the Texas Longhorn: An evolutionary history’, *Environmental History* **21** (2016): 343–63; Recent works in adjacent fields have taken up the topic. M. Derry, D. Haraway, D. Landry, H. Ritvo, S. Swart, K. Guest and M. Mattfeld, ‘Humanimalia roundtable on breed’, *HUMaNIMALIA* **10**(1) (2018): 5–26.

24 R. Woods, *Herds Shot Round the World: Native Breeds and the British Empire, 1800–1900* (Chapel Hill: University of North Carolina Press, 2017).

25 Hence the present article’s title, which is owed to Eric Wolf, *Europe and the People without History* (Berkeley: University of California Press, 1982).

26 P. Richards, *Indigenous Agricultural Revolution: Ecology and Food Production in West Africa* (London: Hutchinson, 1985); Krätli, ‘Cows who choose’; J. Boutrais, ‘The Fulani and cattle breeds: Crossbreeding and heritage strategies’, *Africa* **77** (1) (2007): 18–35.

27 J. Ford, *The Role of the Trypanosomiases in African Ecology: A Study of the Tsetse Fly Problem* (Oxford: Clarendon Press, 1971); Beinart, ‘African history’, 271-72; D. Bannister, ‘The sorcerer’s apprentice: Sleeping sickness, onchocerciasis, and unintended consequences in Ghana, 1930–60’, *Journal of African History* **62** (1) (2021): 29–57.

28 Brown, ‘From Ubombo to Mkhuzi’.

29 J. Giblin, ‘Trypanosomiasis control in African history: an evaded issue?’, *The Journal of African History* **13** (1) (1990): 59–80. Quote on 65 (italics in original); A.J. Duggan, ‘An historical perspective’, in H.W. Mulligan (ed.), *The African Trypanosomiases* (London: Allen and Unwin, 1970), pp. xli–lxxxviii.

30 J. Ford, ‘Ideas which have influenced attempts to solve the problems of African trypanosomiasis’, *Social Science & Medicine, Part B* **13** (4) (1979): 269–75.

31 Giblin, ‘Trypanosomiasis control’, 59–80.

32 Duggan, ‘An historical perspective’. Duggan only dates the ‘old endemic foci’ of sleeping sickness back to the 1880s. His reason for doing so was found in the credibility of data, dismissing the factuality of traditional lore and lacking other archives from which to cite.

33 Giblin, ‘Trypanosomiasis control’, 63–64. See also n. 66 in L. White, ‘Tsetse visions: Narratives of blood and bugs in colonial Northern Rhodesia, 1931–9’, *Journal of African History* **36** (1995): 219–45.

34 H. Kjekshus, *Ecology Control and Economic Development in East African History: A Case of Tanganyika, 1850–1950* (Berkeley: University of California Press, 1977).

35 R.D. Waller, ‘Tsetse fly in western Narok, Kenya’, *Journal of African History* **31** (1990): 81–101.

36 F.L. Lambrecht, ‘Aspects of evolution and ecology of tsetse flies and trypanosomiasis in prehistoric African environment’, *The Journal of African History* **5** (1) (1964): 1–24; Giblin, ‘Trypanosomiasis Control’, 63–64.

37 E. Russell, *Evolutionary History: Uniting History and Biology to Understand Life on Earth* (Cambridge: Cambridge University Press, 2011).

38 See E. Hennessy, ‘The molecular turn in conservation: Genetics, pristine nature, and the rediscovery of an extinct species of Galápagos giant tortoise’, *Annals of the Association of American Geographers* **105** (1) (2015): 87–104.

39 On the history of cattle differentiation as reconstructed through genetics, see D.G. Bradley, R.T. Loftus, P. Cunningham and D.E. Machugh, ‘Genetics and domestic cattle origins’, *Evolutionary Anthropology* **6** (3) (1998): 79–86; J. Kim, O. Hanotte, O.A. Mwai, T. Dessie, S. Bashir, B. Diallo, M. Agaba, K. Kim, W. Kwak, S. Sung, M. Seo, H. Jeong, T. Kwon, M. Taye, K-D. Song, D. Lim, S. Cho, H-J. Lee, D. Yoon, S.J. Oh, S. Kemp, H-K. Lee and H. Kim, ‘The genome landscape of indigenous African cattle’, *Genome Biology* **18** (34) (2017).

40 A.J. Duggan, ‘A survey of sleeping sickness in Northern Nigeria from the earliest times to the present day’, *Transactions of the Royal Society of Tropical Medicine and Hygiene* **56** (6) (1962): 439; Nkwam, ‘British medical and health policies’, 1.

41 C. van Onselen, ‘Reactions to rinderpest in southern Africa, 1896–7’, *Journal of African History* **13** (1972): 473–88; P. Phoofolo, ‘Epidemics and revolutions: the rinderpest epidemic in late nineteenth-century southern Africa’, *Past & Present* **138** (1993): 112–43.

42 A.J. Duggan, ‘The British contribution to the fight against African trypanosomiasis’, *Annales de la Societé Belge de Medecine Tropical*, **51** (4–5) (1971): 483–88; J.D. Grischow, ‘K.R.S. Morris and tsetse eradication in the Gold Coast, 1928–51’, *Africa* **76** (2006): 381–401.

43 Quote from Murray et al., ‘Trypanotolerance in cattle,’ 370.

44 T. Brown, ‘Await the Jarga: cattle, disease, and livestock development in colonial Gambia’, *Agricultural History* **90** (2) (2016): 230–46; Coghe, ‘A New Pastoral Frontier’.

45 Gambian National Archives, Banjul, The Gambia (hereafter GNA), VET 1/13, Folder ‘African Staff Training,’ Files 16, 40.

46 D. Perfect, ‘Jawara, Sir Dawda Kairaba’, *Oxford Dictionary of National Biography*.

47 D.K. Jawara, *Kairaba* (West Sussex: Domtom Publishing, 2009), p. 169.

48 GNA, ARP 3/11, *Summary of the Proceedings of the 11*^*th*^ *Conference of the Protectorate Chiefs, 1954*; see also, H. Ceesay, ‘Chiefs and protectorate administration in colonial Gambia, 1894–1965’, in B.G. Jallow (ed.) *Leadership in Colonial Africa* (London: Palgrave Macmillan, 2014), pp. 25–53.

49 Jawara, *Kairaba*, pp. 169–78; GNA PUB 15/30, ‘Sir Dawda’s speech at seminar’, *The Gambia News Bulletin*, (Banjul) 3 Nov. 1981, 3.

50 GNA, ARP 25/1, *Annual Report of the Veterinary Department for 1947*; GNA, ARP 25/3, *Annual Report of the Veterinary Department for 1949*.

51 H. Tilley, ‘Ecologies of complexity: tropical environments, African trypanosomiasis, and the science of disease control in British colonial Africa, 1900–1940’, *Osiris* **19** (2004): 21–38.

52 A thorough review of this assessment, still in effect into the 1960s, is presented in Roberts and Gray, ‘Studies. I.’, 212.

53 Earliest commentary by Hall in undocumented report deposited in GNA, VET Folder, File 7.

54 Stewart, ‘The cattle’, 1294–95.

55 GNA, ARP 25/4, *Annual Report of the Veterinary Department for 1950*; GNA, ARP 25/8, *Annual Report of the Veterinary Department for 1954–1955*.

56 GNA, ARP 25/8, *Annual Report of the Veterinary Department for 1954–1955*.

57 T.H. Davey, *Trypanosomiasis in British West Africa* (London: His Majesty’s Stationery Office, 1948); P.A. Buxton, *Trypanosomiasis in Eastern Africa, 1947* (London:His Majesty’s Stationery Office, 1948).

58 T.A.M. Nash, *Tsetse Flies in British West* Africa (London: His Majesty’s Stationery Office, 1948).

59 Nash, *Tsetse Flies*, 72. This was not necessarily the consensus. Stewart called N’Dama ‘the breed *par excellence* for improving inferior cattle’, citing its direct descent from the original African bovine in Egypt. Stewart, ‘The cattle’, 1292

60 D.E. Faulkner and J.D. Brown, *The Improvement of Cattle in British Colonial Territories in Africa* (London: Her Majesty’s Stationery Office, 1953), p. 144.

61 Faulkner and Brown, *The Improvement*, pp. 77–78.

62 Ibid., p. 142.

63 I.L. Mason, *The Classification of West African Livestock*, Commonwealth Bureau of Animal Breeding and Genetics, Technical Communication No. 7 (Farnham Royal, England: Commonwealth Agricultural Bureaux, 1951), p. 6.

64 T. Brown, ‘Await the Jarga’.

65 Jawara, *Kairaba*, pp. 169–73.

66 Jawara served as democratically-elected president until a military coup in 1994.

67 GNA, AGR 26/10, File 45, *10*^*th*^ *and 11*^*th*^ *Annual Report of the Specialist Offices Yundum Agricultural Station, 1965/66 & 1966/67*.

68 Compare this decision to the Cuban introduction of Holstein bloodlines from the Canadian stud bull, published in this journal. Reinaldo Funes-Monzote, ‘The rise and fall of dairy cows in socialist Cuba’, *Global Environment* **9** (2016): 342–75.

69 McIntyre would return to the Glasgow Faculty of Veterinary Medicine in 1968, later serving as Dean from 1974–1977. University of Glasgow, ‘Summer newsletter’, 25 (2010): 7.

70 K. McAulay and P. Yam, ‘Celebrating a tradition of innovation’, *Veterinary Record* **170** (26) (2012): 664–66.

71 W.F.H. Jarrett, F.W. Jennings, W.I.M. McIntyre, W. Mulligan and G.M. Urquhart, ‘Irradiated helminth larvae in vaccination’, *Proceedings of the Royal Society of Medicine* **51**(1958): 743–44; M. Murray, ‘The parasites, predators, places and people I have known: A great adventure’, *Veterinary Parasitology* **81** (1999): 149–58.

72 C. Pierre, *L’elevage dans L’Afrique Occidentale Francaise* (Paris: Gouvernement Général De L'Afrique Occidentale Française, 1906); J. Pollard, ‘Notes on the tsetse-flies of Muri province, Northern Nigeria’, *Bulletin of Entomological Research* **3** (2) (1912): 221; A. Balfour, ‘Animal trypanosomiasis in the Lado (western Mongalla) and notes on tsetse fly traps and on an alleged immune breed of cattle in Southern Kordofan’, *Annals of Tropical Medicine & Parasitology* **7** (1) (1913): 113–24; Stewart, ‘The cattle’, 1291.

73 J.L. Stewart, ‘The West African Shorthorn cattle: their value to Africa as trypanosomiasis-resistant animals’, **63** (1951): 454–57. Quote from 455.

74 R.L. Chandler, ‘Comparative tolerance of West African N’Dama cattle to trypanosomiasis’, *Annals of Tropical Medicine and Parasitology* **46**(2) (1952): 127–34; R.L. Chandler, ‘Studies on the tolerance of N’Dama cattle to trypanosomiasis’, *Journal of Comparative Pathology* **68** (1958): 253–60; L.E. Stephen, ‘Observation on the resistance of West African N’Dama and Zebu cattle to trypanosomiasis following challenge by wild *Glossina morsitans* from an early age’, *Annals of Tropical Medicine and Parasitology* **60** (2) (1966): 230–46.

75 Roberts and Gray, ‘Studies on trypanosome-resistant cattle. II.’, 230.

76 W.I. Morrison, M. Murray and W.I.M. McIntyre, ‘Bovine trypanosomiases’, in M. Ristic and I. McIntyre (eds), *Diseases of Cattle in the Tropics*, Current Topics in Veterinary Medicine and Animal Science Volume 6 (The Hague: Martinus Nijhoff, 1981), pp. 469–97.

77 Murray, Trail and D’Ieteren, ‘Trypanotolerance in cattle’, 379.

78 Murray credited the discovery to McIntyre. See Murray, ‘The parasites’, 151.

79 Murray, Trail and D’Ieteren, ‘Trypanotolerance in cattle’, 379–80; Morrison, Murray and McIntyre, ‘Bovine trypanosomiases’, 478.

80 University of Glasgow, ‘Summer’, 6; E. Boden, ‘Professor Ian McIntyre: Glasgow vet who served in Africa’, *The Independent* (20 June 2008).

81 Murray, ‘The parasites’, 149–58.

82 M. Murray, P.K. Murray and W.I.M. McIntyre, ‘An improved parasitological technique for the diagnosis of African trypanosomiasis’, *Transactions of the Royal Society of Tropical Medicine and Hygiene* **71** (4) (1977): 325–26.

83 ILRAD and ILCA would join to become the International Livestock Research Institute (ILRI).

84 Personal interview with Max Murray, 22 Aug. 2011.

85 Murray and Trail, ‘Comparative epidemiology’, 623–24.

86 ILCA, *Trypanotolerant Livestock*.

87 M. Murray, D.J. Clifford, G. Gettinby, W.F. Snow and W.I.M. McIntyre, ‘Susceptibility to African trypanosomiasis of N’Dama and Zebu cattle in an area of *Glossina morsitans submorsitans* challenge’, *The Veterinary Record* **109** (23) (1981): 503–10.

88 M. Murray, J.C.M. Trail and G.D.M. D’Ieteren, ‘Trypanotolerance in cattle and prospects for the control of trypanosomiasis by selective breeding’, *Revue Scientifique et Technique (International Office of Epizootics)* **9** (1990): 369–86.

89 GNA, PUB 15/30, *The Gambia News Bulletin*, 29 Jan. 1982; GNA PUB 15/30, *The Gambia News Bulletin*, 2 April 1982; GNA, PUB 15/30, *The Gambia News Bulletin*, 21 July 1982; Murray, Trail and Grootenhuis,‘Trypanotolerant livestock’.

90 A. Agyemang, K.A. Fall and A. Drucker, ‘Reducing poverty and food insecurity among poor livestock keepers in West and Central Africa through community-based management of indigenous livestock resources and their environments’ (Banjul, n/d).

91 GNA, PUB 15/30, ‘The Gambia base for Ndama research’, *The Gambia News Bulletin*, 11 March 1981; ‘Sir Dawda’s speech’, 3.

92 GNA, PUB 15/30, *The Gambia News Bulletin* 1 April 1987; Boden, ‘Professor’.

93 For a sample, K. Agyeman, R.H. Dwinger, A.S. Grieve and M.L. Bah, ‘Milk production characteristics and productivity of N’Dama cattle kept under village management in The Gambia’, *Journal of Dairy Science* **74** (1991): 1599–608; J.R. Claxton, P. Leperre, P. Rawlings, W.F. Snow and R.H. Dwinger, ‘Trypanosomiasis in cattle in Gambia: Incidence, prevalence and tsetes challenge’, *Acta Tropica* **50** (1992): 219–25; P. Itty, P. Ankders, J. Zinsstaag, S. Trawally and K. Pfister, ‘Productivity and profitability of sheep production in The Gambia: Implications for livestock development in West Africa’, *Zeitschrift fùr ausländische Landwirtschaft* **36** (2) (1997): 153–72; S. Osaer, b. Goossens, I. Jeffcoate and P. Holmes, ‘Effects of *Trypanosoma congolense* and nutritional supplements in Djallonké ewes on live weight during pregnancy, post partum weight, haematology parameters and lamb performance’, *Research in Veterinary Science* **65** (1998): 65–69; D. Faye, S. Osaer, B. Goossens, J. Van Winghem, P. Dorny, V. Lejon, B. Losson and S. Geerts, ‘Susceptibility of trypanotolerant West African Dwarf goats and F1 crosses with the susceptible Sahelian breed to experimental *Trypanosoma congolense* infection and interactions with helminth infection and different levels of diet’, *Veterinary Parasitology* **108** (2002): 117–36; S. Geerts, S. Osaer, B. Goossens and D. Faye, ‘Trypanotolerance in small ruminants of sub-Saharan Africa’, *Trends in Parasitology* **25** (3) (2008): 132–38.

94 L. Dempfle and J. Jaitner, ‘Case study about the N’Dama breeding programme at the International Trypanotolerance Centre (ITC) in The Gambia’, in *ICAR Technical Series: Developing Breeding Strategies for Lower Input Animal Production Environments* (Rome, 2000), pp. 347–354; N.A. Bosso, N. Corr, M. Njie, A. Fall, E.H. van der Waaij, J.A.M. van Arendonk, J. Jaitner, L. Dempfle and K. Agyemang, ‘The N’Dama cattle genetic improvement programme: a review’, *Animal Genetic Resources* **40** (2007): 65–69; O.F. Olaniyan, ‘Sustaining N'Dama cattle for the resource-poor farmers in The Gambia’, *Bulletin of Animal Health and Production in Africa* **63** (1) (2015): 83–92.

95 ITC was rebranded in 2016 as the West African Livestock Innovation Centre (WALIC), www.walic-wa.org.

96 J. Ford, ‘Ideas which have influenced attempts to solve the problems of African trypanosomiasis’, *Social Science & Medicine* **13B** (1979): 269–75. Quote on 269.

97 D.Ouédraogo, A. Soudré, B. Yougbaré, S. Ouédraogo-Koné, B. Zoma-Traoré, N. Khayatzadeh, A. Traoré, M. Sanou, G. Mészáros, P.A. Burger, O.A. Mwai, M. Wurzinger and J. Sölkner, ‘Genetic improvement of local cattle breeds in West Africa: a review of breeding programs’, *Sustainability* **13** (2021): 2124; K. Marshall, ‘Optimizing the use of breed types in developing country livestock production systems: a neglected research area’, *Journal of Animal Breeding and Genetics* **131** (5) (2014): 329–40; K. Marshall, J.P. Gibson, O. Mwai, J.M. Mwacharo, A. Haile, T. Getachew, R. Mrode and S.J. Kemp, ‘Livestock genomics for developing countries – African examples in practice; *Frontiers in Genetics* **10** (2019): 297; K. Agyemang and A. Fall, ‘Genetic improvement in medium to low-input systems of animal production – experiences to date: the West African experience’, *7*^*th*^ *World Congress on Genetics Applied to Livestock Production* (Montpellier, 2002).

98 Naessens, Grab and Sileghem, ‘Identifying the mechanisms’, 98.

99 James Giblin dismissed disease tolerance as an inherited trait in N’Dama by citing studies that could not confirm it, and omitting the rest. Giblin, ‘Trypanosomiasis control’, 63–64.

100 For a great example, see V.A. Finch and D. Wester, ‘Cattle colors in pastoral herds: natural selection or social preference?’, *Ecology* **58** (6) (1977): 1384–92.

101 Boutrais, ‘The Fulani’.

102 Murray, Trail, and D’Ieteren, ‘Trypanotolerance in cattle’.

103 Kim et al, ‘Cattle genome-wide analysis’, 13.

104 A.R. Freeman, C.M. Meghen, D.E. MacHugh, R.T. Loftus, M.D. Achukwi, A. Bado, B. Sauveroche and D.G. Bradley, ‘Admixture and diversity in West African cattle populations’, *Molecular Ecology* **13** (2004), 3477–87.

105 G.E. Roelants, I. Tamboura, D.B. Sidiki, A. Bassinga and M. Pinder, ‘Trypanotolerance. An individual not a breed character’, *Acta Tropica* **40** (1982): 99–104; C.A. Morris, ‘A review of genetic resistance to disease in *Bos taurus* cattle’, *The Veterinary Journal* **174** (2007): 481–91; R.B. Dolan, ‘Genetics and trypanotolerance’, *Parasitology Today* **3** (5) (1987): 138-139; E.H. van der Waaij, O. Hanotte, J.A.M. van Arendonk, S.J. Kemp, D. Kennedy, A. Gibson and A. Teale, ‘Population parameters for traits defining trypanotolerance in an F2 cross of N’Dama and Boran cattle’, *Livestock Production Science* **84** (2003): 219–30; D. Berthier, M. Peylhard, G-K. Dayo, L. Flori, S. Sylla, S. Bolly, H. Sakande, I. Chantal and S. Thevenon, ‘A comparison of phenotypic traits related to trypanotolerance in five West Africa cattle breeds highlights the value of shorthorn taurine breeds’, *PLoS ONE* **10** (5) (2015).

106 As an example, see P.W. Paling and R.H. Dwinger, ‘Potential of trypanotolerance as a contribution to sustainable livestock production in tsetse affected Africa’, *Veterinary Quarterly* **15** (2) (1993): 60–67.

107 Giblin, ‘Trypanosomiasis control’, p. 59.

